# Improvements in Dentistry

**Published:** 1874-05

**Authors:** Samuel Welchens


### ARTICLE V.

### *Improvements in Dentistry.*

There was a time when artificial dentures possessed real value. They were mounted upon gold and silver plates, and regarded by the wearer as quite comfortable and serviceable; then, plain plate teeth were used, and mounted on plates that were cut out so as to be simply wide enough to cover the maxillary ridge. Where an entire denture was needed, spiral springs were used to keep the plates firm to the gum.

This work, though good in its day, was somewhat troublesome, both to the dentist and the patient. Wax being used for impressions, the fit, of course, was approximate, and the benefit to the patient about the same way, until custom overcame the difficulty. At a subsequent period, however, improvements were introduced, and with the advent of the air-chamber and gum-teeth, we imagined the advancement complete. Rims on the plates, both swaged and turned down, were added to the general tenure of this work, and block teeth, continuous gum work, and the various methods of soldering to avoid warping, were among the more immediate improvements.

The laboratory, then, became the centre of science in the profession, and our best men emulated each other in raising this branch of the profession to the highest standard of artistic skill. It was science in art, and constituted an era in the profession that should never have been suffered to be superseded. The downfall of this art started with the advent of casting plates from the baser metals, and the use of rubber and other vegetable substances.

The very simplicity of this latter kind of work, and the ease with which it is constructed, drove science out of the laboratory, and reduced the mechanical branch of the profession to the level of a trade or handicraft. So far, therefore, as the improvements in this department are concerned, the progress has been backwards from a scientific stand-point, and it behooves us all to see to it, that the operative branch through this progression of appliances and new discoveries, does not meet with the same disaster, thus making the last blunder worse than the first. We think we see symptoms of this already in the increase of the use of the baser metals in foils and amalgams, and the various cements and stuffings which are too readily resorted to, where a more complicated or permanent operation should be performed. Is there not some cause here to fear that that nice artistic manipulation of pure gold will be forced to surrender to the overpowering advance of those cheaper materials for filling teeth?

The new mode of operating upon the natural teeth with the appliances now in vogue, leads the mind into a similar channel of thought, with well grounded fears for the future in that department of our specialty.

With the rubber dam, burring engines, and improved patent pluggers of recent date, it does not require much more talent or skill to become a good, even a first-rate operator in filling teeth, than it did several years ago to put up first-class dentures on the rubber base. The most difficult part of the operation to manage is to adjust the rubber dam. When that is properly done, an ordinary individual, with but a low degree of mechanical ingenuity and but partially expert in working gold, can perform very difficult operations and bring them up to what is termed a first-class standard, when the same parties could with difficulty reach mediocrity in the old method of practice.

Here then is a source of solicitude, lest what we now look upon as the highest attainments of skill be lowered both in value and excellence, by comparatively ignorant and irresponsible men.

It is not our purpose to disparage these improvements, or to underrate the operations which are so well performed by scientific men. But is it not apparent that since the profession has been benefitted by those appliances, what may be termed good operators are far more numerous, whilst earnest, thinking, reading and scientific men are rather on the decline?

But it may be asked, what have we to fear from all this? Why just this. The profession will be literally run down with good superficial operators, who care more for their own pockets than the status of the profession, and it will be dragged down to the level of a common mechanical pursuit. Or, in other words, the mechanical element will be highly cultivated, and the competition arising therefrom, as in the rubber work, will reduce the valuation of sueh work, the intellectual element will become stagnant, the qualifications of the dentist will be meagre and low and unable to reach the true genius of the profession as a branch of the healing art.

As a profession, we want first-class operators. We also need all the appliances we can get to facilitate our operations; but at the same time we must give heed to and appreciate old and well-tried principles, and thus guard and develop that balancing intellectual power which gives the mind scope and discrimination to comprehend the complications of disease, so as to be able fully to meet every case with a due sense of that responsibility which should characterize a member of an honorable profession.

This, of course, will admit of no cutting away of old principles, or even the old method of practice. But it will call into requisition every element of improvement, and lead men to value more highly those primitive principles which served as stepping-stones to higher and more advanced stages of excellence.

There is a majesty in the moral force of a scientific profession that should be the chief safe-guard to its dignity and usefulness, and that should dictate the rule of action in the case of any man who would excel as a practitioner. This does not consist alone in the superiority of this or that operator's work—his own sense of his own excellence, nor of the amount of "*blowing*" he may be capable of. It is the force and power of the quality of usefulness he may possess, in rendering himself useful to the public, with a reciprocal appreciation, and without any further effort to please than simply to *perform well a present duty*, rather than that any man should indulge in this preposterous self-laudation.

The dental profession, like the medical profession is an untiring and ceaseless research for *truth*. In every instance where pathological conditions are present, and their locality and extent are determined by symptoms alone, every step and movement made in the diagnosis is a search for the truth. If, therefore, there is a deficieney here, and the individual who adopts our profession is superficial in those principles, no matter how meritorious his mechanical manipulations may be, *he is a quack*, and no amount of good work or self-praise will extricate him from that unenviable position.—*Penn. Journal of Dental Science.*

---

